# Event and Comment

**Published:** 1913-11-15

**Authors:** 


### DENTAL REGISTER.

Vol. LXVII. November 15, 1913. No. 11

### EVENT AND COMMENT.

THE PROGRESSIVE CLINIC. The difficulty of giving a clinic before a large dental society so that any considerable number of those who may desire to see it can do so with any degree of satisfaction, has caused some inventive genius to suggest what is called the "Progressive Clinic." The audience is divided into sections corresponding to the number of clinicians and the size of the audience, and each member of a section is given a number which entitles him to see a given clinic at a given time. The clinician is allowed so much time to explain his clinic to a section which then moves on and another section is brought up to replace the one just served, and the clinician goes through his clinic again to a new crowd. The scheme is a good one in many respects, as the time is limited it compells the clinician to have his clinic in the best possible form to present it intelligently and completely while his section is before him. If he is slow or bungling the bell rings and he has made a failure. Then again, by this means every one has a chance to witness every clinic given, and no one can monopolize any clinician or disconcert him by asking a lot of fool questions, as the clinician has no time to answer them. The idea is good from the standpoint of the observer, but from our experience on two occasions it is hard on the clinician. Talking the same thing over nine times, and for fifteen minutes each time in rapid and uninterrupted succession is more of an undertaking than any ordinary person can endure without serious physical exhaustion, and from the clinicians standpoint it is extremely unsatisfactory because it must be done so methodically and hurriedly.

PROGRAMME SUGGESTION. As the time is again on for active dental society work, the committees having such matters in charge are racking their brains to secure some novelty for their programmes that will attract larger attendance and increase the general interest in the meeting. We have no inclination to suggest subjects for discussion, as there are many combinations of new and old subjects that need reviewing and further discussion. The prevailing method at this time for securing a large attendance seems to be to invite some illustrious speaker or clinician to entertain or edify, and to solicit attendance by resorting to all sorts of advertising schemes, which at times, resemble the method of the quack doctor or circus posters. The idea seems to be abroad that the announcement must be greatly exaggerated to excite the interest of the members. A recent appeal by one of our State societies calls attention to the fact that this particular society was the largest society in the world, and that no one could afford to miss its meeting or neglect to join it, all of which may be true, but isn't it a bit unprofessional to put it in any such light? Recently we received an announcement of an exhibit of dental supplies that was probably truthful in its statement, but it was printed in the form of a large folder that was intended to impress because of its typographical style. It was not modest or genteel as it should be either in style or statement. We couldn't help wondering when we saw it what kind of wedding cards the man who got that up would send out, or if his wife would use visiting cards of this ostentatious character. We have several times received reminders of approaching dental meetings printed on postal cards with a ludicrous charicature to embelish and emphasize the importance of the occasion and attract attention. As a question of ethics we don't know just where we should draw the line on these things, but it always shocks our sense of propriety to see things done in this irreverent and careless manner. We are not prudish, in fact we enjoy good and wholesome humor, but we believe it should not be so publicly flaunted that it may create the impression of triviality or vulgarity in the thoughtless public mind. Ours is a serious calling and there is a professional dignity attached that needs to be conserved. Won't some dental society programme committee take this matter of programme design and invitation up in a more thoughtful manner, and let us see what the result of a simple and dignified announcement gotten out in good taste, not only as to contents, but with due regard to its typographical appearance and its ethical character would accomplish? I believe we can maintain our own ethical ideals by giving needed attention to some of these details of professional activities and at the same time better command the respect of the lay public.

